# A nationwide survey on Marinesco-Sjögren syndrome in Japan

**DOI:** 10.1186/1750-1172-9-58

**Published:** 2014-04-23

**Authors:** Masahide Goto, Mari Okada, Hirofumi Komaki, Kenji Sugai, Masayuki Sasaki, Satoru Noguchi, Ikuya Nonaka, Ichizo Nishino, Yukiko K Hayashi

**Affiliations:** 1 Department of Child Neurology, National Center Hospital, National Center of Neurology and Psychiatry, Tokyo, Japan; 2 Department of Pediatrics, Hitachiomiya Saiseikai Hospital, Ibaraki, Japan; 3 Department of Neuromuscular Research, National Institute of Neuroscience, National Center of Neurology and Psychiatry, Tokyo, Japan; 4 Department of Clinical Development, Translational Medical Center, National Center of Neurology and Psychiatry, Tokyo, Japan; 5 Department of Neurophysiology, Tokyo Medical University, Tokyo, Japan

**Keywords:** Marinesco-Sjögren syndrome (MSS), *SIL1*, Founder effect, Cataracts, Intellectual disability, Ataxia, Rimmed vacuolar myopathy

## Abstract

**Background:**

Marinesco-Sjögren syndrome (MSS) is an autosomal recessive multisystem disorder characterized by the tetralogy of cerebellar ataxia, congenital cataracts, intellectual disability, and progressive muscle weakness due to myopathy. MSS is extremely rare, and its clinical, pathological, and genetic features are not yet fully understood.

**Methods:**

We conducted a nationwide, questionnaire-based survey on MSS in Japan and carefully reviewed the medical records of 36 patients suspected of having this disease. In addition, pathological examinations of muscles, sequence and haplotype analysis in *SIL1* were performed.

**Results:**

The patients had been examined between the ages of 2 and 52 years. Delayed psychomotor development and cataracts from early childhood were observed in all patients, whereas no life-threatening events were observed. Mutations in *SIL1* were identified in 24 of the 27 patients tested, and 43 of the 48 chromosomes possessed the *SIL1* c.936dupG (p.Leu313fs) mutation. The haplotype analysis revealed that 31 of the 32 chromosomes (96.9%) with the c.936dupG mutation had the same haplotype.

**Conclusions:**

The results of haplotype analysis suggested the presence of a founder effect. The clinical features of patients without *SIL1* mutations were indistinguishable from those with *SIL1* mutations, suggesting the genetic heterogeneity of MSS.

## Background

Marinesco-Sjögren syndrome (MSS; OMIM 248800) is an autosomal recessive multisystem disorder clinically characterized by the tetralogy of cerebellar ataxia, congenital cataracts, intellectual disability, and progressive muscle weakness due to myopathy [1-3]. Additional clinical features, including short stature, hypergonadotropic hypogonadism [4], and strabismus [5], are also observed. Mutations in *SIL1* (Gene ID: 64374) were reported to be causative for MSS [6,7]. This gene encodes SIL1, also known as BiP-associated protein (BAP), which is an endoplasmic reticulum (ER)-resident protein. Bip is an HSP70 chaperone family member located in the ER, and plays a key role in protein quality control. SIL1 regulates the ATPase cycle of BiP for proper protein folding [8,9]. SIL1-deficient woozy mutant mice exhibit progressive ataxia caused by the loss of Purkinje cells via ER stress [10].

MSS is an extremely rare disease, and very few cases have been reported. In this study, we performed a nationwide, questionnaire-based survey on MSS with the aim of characterizing its prevalence, clinical features, natural history, muscle pathological findings, and mutation status.

## Methods

All clinical materials used in this study were obtained for diagnostic purposes with written informed consent. All surveys and experiments performed in this study were approved by the Ethical Committee of the National Center of Neurology and Psychiatry.

### The nationwide, questionnaire-based survey

To elucidate the clinical characteristics of MSS, we conducted a nationwide, questionnaire-based survey in Japan. The first set of questionnaires, which focused on the experience of treating patients suspected of having MSS, was sent to a total of 5,452 Japanese specialists for neurology or pediatric neurology. The second set of questionnaires, which focused on the clinical information of patients with suspected MSS, was sent to their attending physicians. These patients’ medical records were carefully reviewed by 2 of our specialists (M.G., H.K.).

### Histochemistry

Biopsied muscle specimens were flash-frozen in isopentane cooled in liquid nitrogen. Transverse serial frozen sections of 10 μm in thickness were subjected to various types of histochemical staining, including hematoxylin and eosin (H&E), modified Gomori-trichrome (mGT), and ATPases. We obtained biopsied skeletal muscles from a total of 17 unrelated patients clinically suspected of having MSS.

### Sequence analysis of *SIL1*

Genomic DNA was extracted from either frozen muscle or peripheral blood lymphocytes using standard protocols. The PCR primers were designed to amplify all the exons of *SIL1* together with their flanking intronic regions. The primer sequences are available upon request. Direct sequencing was performed using the BigDye Terminator v3.1 Cycle Sequencing system and an ABI3100 automated Genetic Analyzer (Applied Biosystems, Foster City, CA). The sequence data obtained were analyzed using the SeqScape (Applied Biosystems) program and compared with the genomic sequence of *SIL1* in the database (NM_022464). Genetic analysis was performed on DNA from 27 unrelated patients and the parents of 3 of them. Two hundred control chromosomes from healthy individuals were examined for each novel mutation in *SIL1* by direct sequencing.

### Haplotype analysis of *SIL1*

For the haplotype analysis of the *SIL1* genomic region, we performed direct sequencing of the following 11 common single nucleotide variants (SNVs) in the Japanese population: rs11748097, rs929775, rs10045761, rs1433008, rs11958050, rs7717375, rs7722413, rs3763016, rs6596456, rs700629, and rs12653845 (http://www.ncbi.nlm.nih.gov/SNP/). Samples from 21 patients homozygous for the common c.936dupG mutation, the parents of 3 patients, and 92 control Japanese individuals were analyzed.

## Results

### Patients

A total of 1,875 responses (34.4% response rate) were received to the first set of questionnaires. The second set of questionnaires was sent to a total of 37 attending physicians (2.0%) who had treated patients suspected of having MSS. The detailed clinical records of a total of 36 patients were carefully reviewed. The shortfall in the number of patients was due to an overlap of 1 case.

### Sequence analysis of *SIL1*

Frozen muscle or peripheral blood lymphocytes were obtained from 27 of the 36 patients suspected of having MSS, for genetic analysis. Mutations in *SIL1* were identified in 24 out of the 27 patients. Twenty-one patients were homozygous for the previously reported c.936dupG (p.Leu313fs) mutation in exon 9. Patient 4 was homozygous for the previously reported c.603_607del5 (p.Glu201fs) mutation in exon 6 [11]. Patient 12 was homozygous for the previously reported c.331C > T (p.Arg111X) mutation in exon 4 [6,7]. Patient 17 was a compound heterozygote for the novel c.617_618TC > AA (p.Leu206Glu) mutation in exon 6 and the c.936dupG (p.Leu313fs) mutation in exon 9 [12]. All mutations except for p.Leu206Glu are predicted to induce premature termination. Mutation of Leu206, which is highly conserved among species, was predicted to exert a deleterious impact on protein function by the Polymorphism Phenotyping v2 (PolyPhen-2; http://genetics.bwh.harvard.edu/pph2/) and the Sorting Intolerant From Tolerant software (SIFT: http://sift.bii.a-star.edu.sg/). None of these nucleic acid changes were found in the 200 chromosomes from healthy Japanese controls or in the Japanese Single Nucleotide Polymorphisms database (http://snp.ims.u-tokyo.ac.jp/index_ja.html).

### Haplotype analysis of *SIL1*

The *SIL1* c.936dupG mutation was identified in 43 of the 48 chromosomes (89.6%) in our cohort. The patients carrying this mutation were reported from different areas in Japan. To determine whether this was a result of a founder effect, we performed a haplotype analysis using 11 SNVs within or close to *SIL1*. The results revealed that 31 of the 32 chromosomes (96.9%) with the c.936dupG mutation had the same haplotype (P1-P24, Table 1). This haplotype was only found in 18 of the 184 chromosomes (9.8%) from the control group, suggesting a founder effect, although 1 chromosome from Patient 1 had a different haplotype.

**Table 1:** Haplotype analysis

**rs No**	**rs11748097**	**rs929775**	**rs10045761**	*/#	**rs1433008**	**rs11958050**	**rs7717375**	**@**	**rs7722413**	**rs3763016**	**rs6596456**	**rs700629**	**rs12653845**
JPT	A: 0.24	G: 0.18	A: 0.15		C: 0.15	A: 0.76	A: 0.82		C: 0.11	G: 0.11	A: 0.13	A: 0.14	G: 0.11
P1	C/A	T/G	G	N	T	A/G	A	H	T/C	C/G	A	A	T
P2	C	T	G	N	T	A	A	H	T	C	A	A	T
P3	C	T	G	N	T	A	A	H	T	C	A	A	T
P5	C	T	G	N	T	A	A	H	T	C	A	A	T
P6	C	T	G	N	T	A	A	H	T	C	A	A	T
P7	C	T	G	N	T	A	A	H	T	C	A	A	T
P9	C	T	G	N	T	A	A	H	T	C	A	A	T
P10	C	T	G	N	T	A	A	H	T	C	A	A	T
P13	C	T	G	N	T	A	A	H	T	C	A	A	T
P14	C	T	G	N	T	A	A	H	T	C	A	A	T
P15	C	T	G	N	T	A	A	H	T	C	A	A	T
P16	C	T	G	N	T	A	A	H	T	C	A	A	T
P18	C	T	G	N	T	A	A	H	T	C	A	A	T
P20	C	T	G	N	T	A	A	H	T	C	A	A	T
P21	C	T	G	N	T	A	A	H	T	C	A	A	T
P24	C	T	G	N	T	A	A	H	T	C	A	A	T
P12	C	T	G	*	T	A	A	N	T	C	A	A	T
P4	C	T	G	#	T	A	A	N	T	C	G	C	T
P25	C/A	T/G	G/A	N	T/C	A/G	A/G	N	T	C	G	C	T/G
P26	C/A	T/G	G/A	N	T/C	A/G	A/G	N	T	C	G	C	T/G
P27	C	T	G	N	T	A	A	N	T	C	G	C	T

@: c.936dupG, *: c.331C > T, #: c.603_607del, JPT: Japanese frequency.

N: Normal, H: Homozygous.

### Clinical features

Table 2 and Additional file 1: Table S1 show a clinical summary of the patients in our series.

**Table 2:** Clinical summary of patients

***SIL1 *** **mutations**			**Positive (n = 24)**	**Negative (n = 3)**	**Not examined (n = 9)**
Ocular involvements	Cataracts		24/24 (100%), 2y-6y	3/3 (100%)	9/9 (100%)
	Strabismus		10/18 (56%)	1/3 (33%)	5/7 (71%)
Motor functions	Muscle weakness		21/22 (95%), 2y-52y	3/3 (100%)	9/9 (100%)
	Development	Head control	21/21 (100%), 4 m-18 m	5 m-8 m	4 m-7 m
		Sit	20/20 (100%), 10 m-36 m	12 m-18 m	12 m-36 m
		Stand with support	16/20 (80%), 1y-4y	15 m, 24 m	15 m-6y
		Walk with support	16/20 (80%), 2y-22y	2/2 (100%) 15 m, 24 m	3/3 (100%) 15 m-6y
	Loss of ambulation		5/16, 13y-28y		
Cerebellar signs	Hypotonia		21/24 (88%)	3/3 (100%)	9/9 (100%)
	Ataxia		16/24 (67%), 2y-52y	2/3 (100%)	6/8 (75%)
	Nystagmus		11/24 (46%), 2y-45y	0/3	5/8 (63%)
	Dysarthria		8/24 (33%), 2y-48y	2/3 (67%)	4/9 (44%)
Psychomotor delay			20/22 (91%), IQ(DQ):24-100	3/3 (100%)	9/9 (100%)
Hypogonadism			3/8 (38%)	0/1	2/3 (67%)
Skeletal abnormalities	Short stature		12/18 (67%),	1/3 (33%)	3/8 (38%)
	Spinal deformities		8/22 (36%)	1/3 (33%)	3/7 (43%)
	Flat foot		7/22 (32%)	0/3	1/7 (14%)
	Short fingers		5/22 (23%)	0/3	2/8 (25%)
Others	Serum CK (IU/L)		28-2000	144-3010	95-600
	Cerebellar atrophy		19/19 (100%)	3/3 (100%)	9/9(100%)
	Rimmed vacuoles in muscles		16/16 (100%)	0/1	2/6 (33%)

The age at which the examination was performed in the 24 patients (10 men and 14 women) with *SIL1* mutations varied from 2 to 52 (mean = 20.1 ± 18.1) years. Bilateral cataracts requiring prompt surgical intervention had appeared and rapidly progressed in all 24 patients at the mean age of 3.5 ± 1.2 years. Strabismus was also observed in 55.6% (10/18) of the patients. Cerebellar signs included hypotonia (21/24; 88%), ataxia (16/24; 67%), nystagmus (11/24; 46%), and dysarthria (8/24; 33%) were seen. Brain MRI demonstrated marked atrophy of the cerebellum, particularly the vermis, in all the patients examined (19/19). Mild to moderate intellectual disability, diagnosed by intelligence quotient/developmental quotient between 35 and 70, was seen in 91% (20/22) of the patients. Acquisition of meaningful words occurred at the age of 2.0 ± 0.8 years, and most of the patients had required special-needs education. Muscle weakness was observed in 95% (21/22) of the patients, with delays in motor milestones. Head control was first seen in all the patients at a certain time point between 4 and 18 (mean = 7.8 ± 3.7) months, and sitting at a certain time point between 10 and 36 (mean = 20.0 ± 8.7) months. Eighty percent (16/20) of the patients could stand with support at a certain point between the ages of 1 and 4 years, and walk with support at a certain point between the ages of 2 and 22 (mean = 5.8 ± 2.6) years; however, none of the patients acquired the ability to walk independently. Muscle weakness was slowly progressive and predominantly in the proximal muscles, with the patients becoming wheelchair-bound at a certain time point between the ages of 13 and 28 (mean = 17.4 ± 6.3) years. Serum creatine kinase levels were normal to moderately elevated (28–2000, mean = 389 ± 464; normal < 200 IU/L). Short stature (< -2 SD) was seen in 67% (12/18, mean = -3.6 SD) of the patients, and spinal deformity (8/22; 36%), flat foot (7/22; 32%), and short fingers (5/22; 23%) were also reported. Hypogonadotropic hypogonadism was seen in 3 of 8 (38%) patients (1 with microtestis, 2 with amenorrhea). No marked clinical differences were observed among patients with different *SIL1* mutations. No patient had cardiac and respiratory problems.

The 3 patients with no *SIL1* mutation (Patients 25, 26, and 27) and the 9 genetically unexamined patients showed clinical features indistinguishable from the patients with *SIL1* mutations, including cerebellar signs with cerebellar atrophy on brain images, intellectual disability, congenital cataracts, and muscle weakness. Elevation of serum CK levels was also seen in 2 patients (Table 2).

### Pathological findings of skeletal muscles

**Figure 1:**
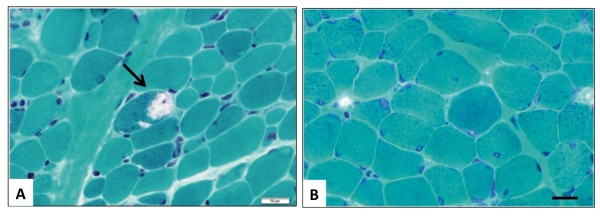
**Modified gomori trichrome stain of the biopsied skeletal muscles.** A muscle from a MSS patient with *SIL1* mutation shows rimmed vacuoles (arrow, **A)**, whereas no vacuole is seen in a patient without *SIL1* mutation **(B)**. Bar = 20 μm.

Biopsied skeletal muscles were obtained from 16 patients with *SIL1* mutations and one patient without (Patient 27). All muscle specimens showed myopathic changes of variation in fiber size and endomysial fibrosis. A few necrotic and regenerating fibers were seen in some patients with *SIL1* mutations. No neurogenic changes, including fiber type grouping and grouped atrophy, were observed in any of the patients. Importantly, scattered rimmed vacuoles (RVs) were seen in all 16 patients with *SIL1* mutations, but not in the patient without (Figure 1).

## Discussion

We conducted a nationwide, questionnaire-based survey to clarify the prevalence, clinical and pathological characteristics, and long-term course of MSS in Japanese patients. The total number of patients with MSS was only 36.

From a clinical point of view, it is important to carry out careful ophthalmological examination of MSS patients at a young age if visual acuity is to be preserved, as the cataracts characteristic of MSS usually appear abruptly and develop rapidly from an early age [13,14]. Indeed, all of the patients in our present series required early and prompt surgical intervention. Marked cerebellar atrophy on brain MRI is another characteristic of this disease, however cerebellar ataxia can be difficult to identify, especially in younger patients with muscle weakness. Skeletal muscle weakness is also a prominent characteristic. Almost all the MSS patients with *SIL1* mutations in this series had muscle weakness initially noticed as a delayed motor milestone, which was detected at an earlier age than cataracts, as reported previously [3,15,16]. Regarding muscle biopsy, myopathic changes, including RV formation are a characteristic of patients with *SIL1* mutations. RVs are not disease-specific, and are often seen in adult-onset chronic myopathies such as inclusion body myositis, distal myopathy with RVs, oculopharyngeal muscular dystrophy, and myofibrillar myopathies. They are rarely observed, however, in childhood-onset myopathies. The presence of RVs in muscle biopsy tissue can be helpful in formulating an early diagnosis of MSS, allowing the ophthalmologist to perform surgery for cataracts to prevent total visual loss. The life prognosis of MSS appears to be comparatively good, as respiratory, cardiac, and swallowing functions are well preserved, even in the patients who are over 50 years of age.

Most of the reported *SIL1* gene mutations have been predicted to induce premature termination and loss of function of SIL1. Based on a putative model of SIL1-BiP interaction, the C-terminal 5 amino acids of SIL1 are thought to play a key role in its association with BiP [17]. This concept is further strengthened by the fact that the p.Arg111X, p.Glu201fs, and p.Leu313fs mutations cause the generation of SIL1 proteins lacking the C-terminal region. Complete loss of function due to nonsense-mediated mRNA decay should also be considered. On the other hand, Leu206 in exon 6 is well preserved among species, and the novel nonsynonymous mutation p.Leu206Glu is predicted to exert a deleterious impact on protein function by both SIFT and PolyPhen2. The c.936dupG (p.Leu313fs) mutation in *SIL1*, which was first reported from Japan [12], is highly common in Japanese MSS patients. Haplotype analysis revealed that whereas 96.9% of chromosomes from MSS patients possessing the c.936dupG mutation had the same haplotype, less than 10% of the chromosomes of the controls did so, suggesting a founder effect.

The results of this study also strongly suggest the genetic heterogeneity of MSS. Three of the 27 patients (11.1%) had no *SIL1* mutation, but demonstrated the cardinal features of MSS, including congenital cataracts, ataxia, intellectual disability, and myopathy. We could not exclude the possibility of the mutation occurred in the promoter or other non-coding region of *SIL1* in these 3 patients. Previous reports also showed that approximately one-half of the MSS patients were genetically diagnosed as MSS from mutations in *SIL1*[7,16]. The absence of RVs in the muscle biopsy tissue of one patient with no *SIL1* mutation suggests the existence of a different disease mechanism(s) in such patients. Further analysis is required to identify the other causative genes for MSS.

## Conclusions

MSS is an extremely rare disease, but a possible founder effect was present in Japan. The life prognosis of MSS is comparatively good, and early diagnosis is important for prevention of a total visual loss. Other causative genes for MSS can cause indistinguishable clinical features via different disease mechanisms.

## Competing interests

The authors declare that they have no competing of interest.

## Authors’ contributions

MG had full access to all the data in the study and wrote the manuscript; MO performed the mutation analysis; HK participated in analyzing all the clinical data; KS, MS, SN, I Nonaka, and I Nishino were involved in data interpretation and also supervised manuscript preparation. YKH supervised all aspects of the study, including study design, data interpretation, and manuscript preparation. All authors read and approved the final manuscript.

## Supplementary Material

Additional file 1: Table S1 Clinical findings of each patient with or without *SIL1* mutations. Click here for file
