# Supplementary material for: A nationwide survey on Marinesco-Sjögren syndrome in Japan
Source: Orphanet J Rare Dis. 2014 Apr 23;9:58. doi: 10.1186/1750-1172-9-58 (PMC4021608; doi:10.1186/1750-1172-9-58)
Supplement: Additional file 1: Table S1 — Clinical findings of each patient with or without SIL1 mutations. [file 1750-1172-9-58-S1.docx]

Supplemental Table. Clinical findings of each patient with or without *SIL1* mutations

| Patient | Age  (yrs) | Sex | Mutation | Zygosity | Cataracts  (yrs) | Muscle weakness | Walk with  Support (yrs) | Cerebellar signs | Cerebellar atrophy | Intellectual disability | Serum CK | RVs |
| --- | --- | --- | --- | --- | --- | --- | --- | --- | --- | --- | --- | --- |
| P1 | 2 | F | c.937dupG | homo | 2 | Y | 2 | h, a, n, s | Y | normal | 407 | Y |
| P2 | 3 | F | c.937dupG | homo | 2.8 | Y | NA | h | Y | DQ 64 | 95 | Y |
| P3 | 3 | F | c.937dupG | homo | 3 | Y | NY | h | Y | DQ 67 | 203 | Y |
| P4 | 3 | F | c.603_607del5 | homo | Y | Y | NY | h, a | Y | DQ 49 | NA | Y |
| P5 | 4 | F | c.937dupG | homo | 2.5 | Y | NA | h, a | Y | NA | 123 | Y |
| P6 | 4 | F | c.937dupG | homo | 5 | Y | 3.8 | h | Y | decline | 117 | Y |
| P7 | 5 | M | c.937dupG | homo | 2 | Y | 2 | h, a, n, s | Y vermis | DQ 60 | 1,000 | Y |
| P8 | 5 | M | c.937dupG | homo | 4.3 | Y | 2.4 | h, a, n | Y | DQ 50 | 281 | ND |
| P9 | 6 | M | c.937dupG | homo | 6 | Y | N | a | NA | normal | 198 | ND |
| P10 | 8 | F | c.937dupG | homo | Y | Y | NA | h | NA | NA | high | ND |
| P11 | 8 | F | c.937dupG | homo | 3 | Y | N | h | Y | decline | 365 | Y |
| P12 | 11 | M | c.331C>T | homo | Y | Y | Y | h | NA | decline | 2,000 | Y |
| P13 | 12 | F | c.937dupG | homo | 3 | Y | 7 | h, a | Y | decline | 326 | Y |
| P14 | 20 | M | c.937dupG | homo | 6 | Y | 2 | h, a, n, s | Y | IQ 24 | 776 | Y |
| P15 | 23 | M | c.937dupG | homo | 4 | Y | Y | h, a | Y | IQ 57 | 974 | Y |
| P16 | 23 | M | c.937dupG | homo | 2 | Y | 3 | h, a, n, s | Y | decline | 320 | Y |
| P17 | 28 | F | c.937dupG/  c.617_618TC>AA | comp  hetero | 3 | Y | 4 | a, n | Y | IQ 60 | NA | ND |
| P18 | 30 | F | c.937dupG | homo | 3 | Y | 5 | a, n | Y | IQ 48 | 186 | Y |
| P19 | 42 | M | c.937dupG | homo | 3 | Y | 5 | h, a, n, s | Y vermis | decline | 410 | ND |
| P20 | 45 | F | c.937dupG | homo | Y | Y | Y | h, a, n, s | Y | IQ 60 | 46 | ND |
| P21 | 45 | F | c.937dupG | homo | 3 | Y | 10 | h, n | NA | IQ 50 | 129 | Y |
| P22 | 48 | M | c.937dupG | homo | Y | Y | NA | h, s | Y | decline | 120 | ND |
| P23 | 52 | F | c.937dupG | homo | 3 | Y | 10 | h, a | Y | decline | 28 | ND |
| P24 | 52 | M | c.937dupG | homo | Y | Y | 6 | h, a, n, s | NA | IQ 54 | 67 | Y |
| P25 | 3 | F | no mut |  | 0 | Y | 1.3 | N | Y vermis | decline | 371 | ND |
| P26 | 6 | F | no mut |  | 6 | Y | NY | a | Y | DQ 65 | 3,010 | ND |
| P27 | 29 | F | nomut |  | 1 | Y | 2 | a, s | Y vermis | IQ 27 | 144 | N |

homo: homozygous, comp hetero: compound heterozygous, F: female, M: male, Y: yes, N: no, NA: not available, ND: not done, NY: not yet, h: hypotonia, a: ataxia, n: nystagmus, s: cerebellar speech, DQ: developmental quotient, IQ: intelligence quotient, CK: creatine kinase (normal: 51-197 U/L), RVs: rimmed vacuoles, No mut: no mutation in *SIL1*
